# 1,3-Dihydr­oxy-2-methoxy­methyl-9,10-anthraquinone from *Rennellia elliptica* Korth.

**DOI:** 10.1107/S1600536809017607

**Published:** 2009-05-29

**Authors:** Nor Hadiani Ismail, Che Puteh Osman, Rohaya Ahmad, Khalijah Awang, Seik Weng Ng

**Affiliations:** a Faculty of Applied Sciences, Universiti Teknologi MARA, 40450 Shah Alam, Selangor Darul Ehsan, Malaysia; b Department of Chemistry, University of Malaya, 50603 Kuala Lumpur, Malaysia

## Abstract

The title compound, C_16_H_12_O_5_, common name: lucidin ω-methyl ether, exists as a planar mol­ecule (r.m.s. deviation = 0.04 Å). Within the mol­ecule, the 1-hydr­oxy group forms a hydrogen bond to the adjacent carbonyl O atom, and the 3-hydr­oxy group forms a hydrogen bond to the adjacent meth­oxy O atom. The meth­oxy O atom is disordered over two positions of equal occupancy.

## Related literature

The title compound has been isolated from several plants: *Rubia tinctorum* L. (Boldizsar *et al.*, 2004 ▶), *taurina* subsp. caucasica (Ozgen *et al.*, 2006 ▶), *Prismatomeris fragrans (*Kanokmedhakul *et al.*, 2005 ▶), *Crucianella maritima* L. (El-Lakany *et al.*, 2004 ▶), *Rubia wallichiana* Decne (Wu *et al.*, 2003 ▶), *Morinda elliptica* (Ali *et al.*, 2000 ▶; Ismail *et al.*, 1997 ▶; Ismail *et al.*, 2002 ▶), *Ophiorrhiza pumila* (Kitajima *et al.*, 1998 ▶), *Morinda officinalis* How. (Yoshikawa *et al.*, 1995 ▶), *Galiumspurium* var. echinospermon (Koyama *et al.*, 1993 ▶), *Damnacanthus indicus* (Koyama *et al.*, 1992 ▶), *Rubia cordifolia* L. (Vidal-Tessier *et al.*, 1987 ▶), *Faramea cyanea *(Ferrari *et al.*, 1985 ▶), *Morinda parvifolia *(Chang & Lee, 1984 ▶) and *Galium album* (Kupier & Labadie, 1984 ▶).
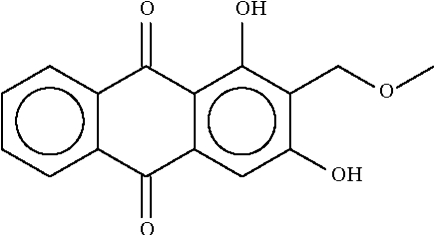


## Experimental

### 

#### Crystal data

C_16_H_12_O_5_
*M* *_r_* = 284.26
Monoclinic, 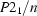

*a* = 4.6725 (1) Å
*b* = 39.685 (1) Å
*c* = 6.9869 (2) Å
β = 107.654 (2)°
*V* = 1234.55 (6) Å^3^
*Z* = 4
Mo *K*α radiation
μ = 0.12 mm^−1^
*T* = 100 K
0.30 × 0.07 × 0.02 mm

#### Data collection

Bruker SMART APEX diffractometer
Absorption correction: none
10046 measured reflections
2825 independent reflections
1888 reflections with *I* > 2σ(*I*)
*R*_int_ = 0.041

#### Refinement

*R*[*F* ^2^ > 2σ(*F* ^2^)] = 0.054
*wR*(*F* ^2^) = 0.156
*S* = 1.01
2825 reflections
201 parameters
4 restraints
H atoms treated by a mixture of independent and constrained refinement
Δρ_max_ = 0.43 e Å^−3^
Δρ_min_ = −0.46 e Å^−3^

### 

Data collection: *APEX2* (Bruker, 2008 ▶); cell refinement: *SAINT* (Bruker, 2008 ▶); data reduction: *SAINT*; program(s) used to solve structure: *SHELXS97* (Sheldrick, 2008 ▶); program(s) used to refine structure: *SHELXL97* (Sheldrick, 2008 ▶); molecular graphics: *X-SEED* (Barbour, 2001 ▶); software used to prepare material for publication: *publCIF* (Westrip, 2009 ▶).

## Supplementary Material

Crystal structure: contains datablocks global, I. DOI: 10.1107/S1600536809017607/tk2447sup1.cif

Structure factors: contains datablocks I. DOI: 10.1107/S1600536809017607/tk2447Isup2.hkl

Additional supplementary materials:  crystallographic information; 3D view; checkCIF report

## Figures and Tables

**Table 1:** Hydrogen-bond geometry (Å, °)

*D*—H⋯*A*	*D*—H	H⋯*A*	*D*⋯*A*	*D*—H⋯*A*
O1—H1*o*⋯O2	0.85 (1)	1.79 (2)	2.557 (2)	150 (3)
O4—H4*o*⋯O5	0.84 (1)	1.77 (2)	2.546 (7)	152 (4)
O4—H4*o*⋯O5′	0.84 (1)	1.77 (2)	2.539 (7)	152 (4)

**Fig. 1.**
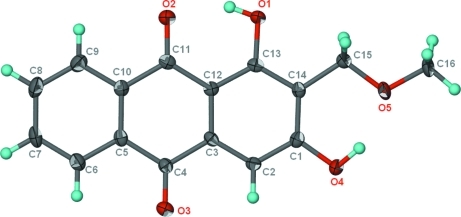
Thermal ellipsoid plot (Barbour, 2001) of the molecule of C16H12O5 at the 70% probability level. Hydrogen atoms are drawn as spheres of arbitrary radius. The disorder is not shown

## supplementary crystallographic information

### Experimental

About 1 kg of the root of *Rennelia elliptica* Korth., which was collected from the Kuala Keniam National Park, Malaysia, was extracted with dichloromethane. The solvent was removed to give a crude material (approx. 10 g) that was fractionated on a chromatography column (60 x 5 cm) packed with silica. The silica had been previously immersed in 4% oxalic acid and then activated by heating to 363 K. The fractions were eluted with hexane–dichloromethane and dichloromethane–methanol in increasing polarity. The fraction eluted with hexane–dichloromethane (2:8 v/v) was purified by thin layer chromatography (2 mm). The product was recrystallized from dichloromethane to furnish yellow crystals. The formulation was established by ^1^H- and ^13^C-NMR spectroscopy.

### Refinement

Hydrogen atoms were placed at calculated positions (C–H 0.95–0.99 Å) and were treated as riding on their parent carbon atoms, with *U*(H) set to 1.2–1.5 times *U*_eq_(*C*). The hydroxy H-atoms were located in a difference Fourier map, and were refined with a distance restraint of O–H 0.84±0.01 Å; their temperature factors were refined.

The methoxy oxygen atom is disordered over two positions, but the occupancy could not be refined. The disorder was assumed to be 50:50. The C–O/C–O' bonds to the aryl group were restrained to within 0.01 Å of each other, as were those to the alkyl group. The anisotropic displacement factors of the primed atom were restrained to those of the umprimed one.

### Crystal data

**Table:**

C_16_H_12_O_5_	*F*(000) = 592
*M**_r_* = 284.26	*D*_x_ = 1.529 Mg m^−^^3^
Monoclinic, *P*2_1_/*n*	Mo *K*α radiation, λ = 0.71073 Å
Hall symbol: -P 2yn	Cell parameters from 1810 reflections
*a* = 4.6725 (1) Å	θ = 3.1–27.9°
*b* = 39.685 (1) Å	µ = 0.12 mm^−^^1^
*c* = 6.9869 (2) Å	*T* = 100 K
β = 107.654 (2)°	Plate, yellow
*V* = 1234.55 (6) Å^3^	0.30 × 0.07 × 0.02 mm
*Z* = 4	

### Data collection

**Table:**

Bruker SMART APEX diffractometer	1888 reflections with *I* > 2σ(*I*)
Radiation source: fine-focus sealed tube	*R*_int_ = 0.041
graphite	θ_max_ = 27.5°, θ_min_ = 1.0°
ω scans	*h* = −5→6
10046 measured reflections	*k* = −51→51
2825 independent reflections	*l* = −9→8

### Refinement

**Table:**

Refinement on *F*^2^	Primary atom site location: structure-invariant direct methods
Least-squares matrix: full	Secondary atom site location: difference Fourier map
*R*[*F*^2^ > 2σ(*F*^2^)] = 0.054	Hydrogen site location: inferred from neighbouring sites
*wR*(*F*^2^) = 0.156	H atoms treated by a mixture of independent and constrained refinement
*S* = 1.01	*w* = 1/[σ^2^(*F*_o_^2^) + (0.0691*P*)^2^ + 1.3159*P*] where *P* = (*F*_o_^2^ + 2*F*_c_^2^)/3
2825 reflections	(Δ/σ)_max_ = 0.001
201 parameters	Δρ_max_ = 0.43 e Å^−^^3^
4 restraints	Δρ_min_ = −0.46 e Å^−^^3^

### Fractional atomic coordinates and isotropic or equivalent isotropic displacement parameters (Å^2^)

**Table:**

	*x*	*y*	*z*	*U*_iso_*/*U*_eq_	Occ. (<1)
O1	0.5327 (4)	0.35893 (4)	0.3196 (2)	0.0173 (4)	
H1o	0.476 (7)	0.3392 (4)	0.338 (5)	0.039 (9)*	
O2	0.2892 (4)	0.31121 (4)	0.4610 (3)	0.0200 (4)	
O3	0.0049 (4)	0.39767 (4)	0.9709 (2)	0.0189 (4)	
O4	0.5244 (4)	0.46858 (4)	0.5956 (3)	0.0202 (4)	
H4o	0.601 (8)	0.4724 (9)	0.503 (4)	0.051 (11)*	
O5	0.777 (3)	0.45857 (15)	0.3246 (15)	0.027 (2)	0.50
O5'	0.694 (3)	0.46028 (15)	0.2862 (15)	0.027 (2)	0.50
C1	0.4569 (5)	0.43535 (6)	0.5813 (3)	0.0144 (5)	
C2	0.3075 (5)	0.42345 (6)	0.7145 (3)	0.0137 (5)	
H2	0.2585	0.4386	0.8052	0.016*	
C3	0.2318 (5)	0.38992 (6)	0.7142 (3)	0.0129 (5)	
C4	0.0737 (5)	0.37804 (6)	0.8575 (3)	0.0135 (5)	
C5	0.0051 (5)	0.34142 (6)	0.8590 (3)	0.0139 (5)	
C6	−0.1291 (5)	0.32923 (6)	0.9985 (4)	0.0183 (5)	
H6	−0.1774	0.3443	1.0900	0.022*	
C7	−0.1917 (6)	0.29523 (6)	1.0034 (4)	0.0217 (5)	
H7	−0.2798	0.2869	1.1001	0.026*	
C8	−0.1268 (6)	0.27307 (6)	0.8677 (4)	0.0229 (6)	
H8	−0.1728	0.2498	0.8707	0.028*	
C9	0.0054 (6)	0.28509 (6)	0.7283 (4)	0.0203 (5)	
H9	0.0490	0.2700	0.6352	0.024*	
C10	0.0745 (5)	0.31922 (6)	0.7240 (3)	0.0146 (5)	
C11	0.2280 (5)	0.33153 (6)	0.5794 (3)	0.0141 (5)	
C12	0.3044 (5)	0.36698 (6)	0.5816 (3)	0.0132 (5)	
C13	0.4545 (5)	0.37961 (6)	0.4486 (3)	0.0129 (5)	
C14	0.5296 (5)	0.41375 (6)	0.4458 (3)	0.0131 (5)	
C15	0.6804 (5)	0.42442 (6)	0.2919 (3)	0.0153 (5)	
H15A	0.8555	0.4097	0.3022	0.018*	0.50
H15B	0.5378	0.4220	0.1551	0.018*	0.50
H15C	0.8858	0.4149	0.3277	0.018*	0.50
H15D	0.5654	0.4157	0.1578	0.018*	0.50
C16	0.8677 (6)	0.47240 (6)	0.1631 (4)	0.0221 (6)	
H16A	0.9308	0.4958	0.1938	0.033*	0.50
H16B	0.6989	0.4716	0.0390	0.033*	0.50
H16C	1.0357	0.4592	0.1459	0.033*	0.50
H16D	0.9262	0.4958	0.1984	0.033*	0.50
H16E	0.7474	0.4711	0.0215	0.033*	0.50
H16F	1.0482	0.4585	0.1853	0.033*	0.50

### Atomic displacement parameters (Å^2^)

**Table:**

	*U*^11^	*U*^22^	*U*^33^	*U*^12^	*U*^13^	*U*^23^
O1	0.0221 (9)	0.0145 (9)	0.0194 (9)	−0.0005 (7)	0.0124 (7)	−0.0022 (7)
O2	0.0264 (9)	0.0162 (8)	0.0209 (9)	−0.0008 (7)	0.0122 (8)	−0.0020 (7)
O3	0.0220 (9)	0.0190 (9)	0.0184 (9)	0.0000 (7)	0.0102 (7)	−0.0021 (7)
O4	0.0303 (10)	0.0136 (8)	0.0205 (9)	−0.0041 (7)	0.0134 (8)	−0.0010 (7)
O5	0.036 (6)	0.0142 (10)	0.043 (3)	0.0027 (18)	0.033 (4)	0.0050 (14)
O5'	0.036 (6)	0.0142 (10)	0.043 (3)	0.0027 (18)	0.033 (4)	0.0050 (14)
C1	0.0138 (11)	0.0130 (11)	0.0156 (11)	−0.0001 (9)	0.0032 (9)	0.0012 (8)
C2	0.0135 (11)	0.0143 (11)	0.0138 (11)	0.0003 (8)	0.0050 (9)	−0.0016 (8)
C3	0.0099 (11)	0.0171 (12)	0.0121 (11)	0.0005 (9)	0.0038 (9)	0.0006 (8)
C4	0.0122 (11)	0.0151 (11)	0.0131 (11)	0.0003 (9)	0.0040 (9)	0.0008 (9)
C5	0.0103 (11)	0.0164 (11)	0.0147 (11)	−0.0009 (8)	0.0032 (9)	0.0023 (9)
C6	0.0180 (12)	0.0196 (12)	0.0189 (12)	0.0006 (10)	0.0082 (10)	0.0024 (9)
C7	0.0217 (13)	0.0231 (13)	0.0230 (13)	−0.0038 (10)	0.0110 (10)	0.0064 (10)
C8	0.0245 (13)	0.0147 (12)	0.0308 (14)	−0.0030 (10)	0.0102 (11)	0.0037 (10)
C9	0.0217 (13)	0.0151 (12)	0.0248 (13)	−0.0005 (9)	0.0083 (11)	−0.0011 (10)
C10	0.0135 (11)	0.0144 (11)	0.0154 (12)	0.0015 (9)	0.0038 (9)	0.0023 (9)
C11	0.0116 (11)	0.0166 (11)	0.0137 (11)	0.0035 (9)	0.0031 (9)	0.0007 (9)
C12	0.0128 (11)	0.0136 (11)	0.0124 (11)	0.0008 (8)	0.0027 (9)	0.0002 (8)
C13	0.0098 (11)	0.0163 (11)	0.0114 (11)	0.0022 (8)	0.0015 (8)	−0.0008 (8)
C14	0.0104 (11)	0.0147 (11)	0.0143 (11)	0.0003 (8)	0.0036 (9)	0.0025 (9)
C15	0.0168 (12)	0.0146 (11)	0.0157 (11)	−0.0002 (9)	0.0068 (9)	0.0001 (9)
C16	0.0273 (14)	0.0184 (12)	0.0257 (14)	−0.0043 (10)	0.0156 (11)	0.0049 (10)

### Geometric parameters (Å, °)

**Table:**

O1—C13	1.349 (3)	C7—C8	1.392 (4)
O1—H1o	0.848 (10)	C7—H7	0.9500
O2—C11	1.249 (3)	C8—C9	1.387 (3)
O3—C4	1.222 (3)	C8—H8	0.9500
O4—C1	1.353 (3)	C9—C10	1.395 (3)
O4—H4o	0.842 (10)	C9—H9	0.9500
O5—C15	1.425 (6)	C10—C11	1.488 (3)
O5—C16	1.431 (6)	C11—C12	1.450 (3)
O5'—C15	1.426 (6)	C12—C13	1.415 (3)
O5'—C16	1.432 (6)	C13—C14	1.401 (3)
C1—C14	1.393 (3)	C14—C15	1.514 (3)
C1—C2	1.404 (3)	C15—H15A	0.9900
C2—C3	1.377 (3)	C15—H15B	0.9900
C2—H2	0.9500	C15—H15C	0.9900
C3—C12	1.412 (3)	C15—H15D	0.9900
C3—C4	1.490 (3)	C16—H16A	0.9800
C4—C5	1.489 (3)	C16—H16B	0.9800
C5—C6	1.396 (3)	C16—H16C	0.9800
C5—C10	1.399 (3)	C16—H16D	0.9800
C6—C7	1.383 (3)	C16—H16E	0.9800
C6—H6	0.9500	C16—H16F	0.9800
			
C13—O1—H1o	107 (2)	C3—C12—C13	117.9 (2)
C1—O4—H4o	105 (2)	C3—C12—C11	121.7 (2)
C15—O5—C16	113.1 (5)	C13—C12—C11	120.4 (2)
C15—O5'—C16	113.0 (5)	O1—C13—C14	117.32 (19)
O4—C1—C14	123.4 (2)	O1—C13—C12	120.8 (2)
O4—C1—C2	115.5 (2)	C14—C13—C12	121.9 (2)
C14—C1—C2	121.1 (2)	C1—C14—C13	118.1 (2)
C3—C2—C1	120.2 (2)	C1—C14—C15	124.9 (2)
C3—C2—H2	119.9	C13—C14—C15	116.92 (19)
C1—C2—H2	119.9	O5—C15—C14	110.1 (3)
C2—C3—C12	120.8 (2)	O5'—C15—C14	109.5 (3)
C2—C3—C4	119.0 (2)	O5—C15—H15A	109.6
C12—C3—C4	120.3 (2)	O5'—C15—H15A	123.2
O3—C4—C5	121.2 (2)	C14—C15—H15A	109.6
O3—C4—C3	121.1 (2)	O5—C15—H15B	109.6
C5—C4—C3	117.73 (19)	C14—C15—H15B	109.6
C6—C5—C10	119.8 (2)	H15A—C15—H15B	108.2
C6—C5—C4	119.1 (2)	O5'—C15—H15C	109.8
C10—C5—C4	121.0 (2)	C14—C15—H15C	109.8
C7—C6—C5	119.9 (2)	O5'—C15—H15D	109.8
C7—C6—H6	120.0	C14—C15—H15D	109.8
C5—C6—H6	120.0	H15C—C15—H15D	108.2
C6—C7—C8	120.5 (2)	O5—C16—H16A	109.5
C6—C7—H7	119.7	O5—C16—H16B	109.5
C8—C7—H7	119.7	H16A—C16—H16B	109.5
C9—C8—C7	119.8 (2)	O5—C16—H16C	109.5
C9—C8—H8	120.1	H16A—C16—H16C	109.5
C7—C8—H8	120.1	H16B—C16—H16C	109.5
C8—C9—C10	120.3 (2)	O5—C16—H16D	107.0
C8—C9—H9	119.9	O5'—C16—H16D	109.5
C10—C9—H9	119.9	H16B—C16—H16D	110.0
C9—C10—C5	119.7 (2)	H16C—C16—H16D	111.3
C9—C10—C11	119.7 (2)	O5'—C16—H16E	109.5
C5—C10—C11	120.6 (2)	H16D—C16—H16E	109.5
O2—C11—C12	121.9 (2)	O5'—C16—H16F	109.5
O2—C11—C10	119.5 (2)	H16D—C16—H16F	109.5
C12—C11—C10	118.62 (19)	H16E—C16—H16F	109.5
			
O4—C1—C2—C3	−179.2 (2)	C2—C3—C12—C11	179.7 (2)
C14—C1—C2—C3	0.5 (3)	C4—C3—C12—C11	0.2 (3)
C1—C2—C3—C12	0.4 (3)	O2—C11—C12—C3	179.3 (2)
C1—C2—C3—C4	179.9 (2)	C10—C11—C12—C3	−1.2 (3)
C2—C3—C4—O3	1.7 (3)	O2—C11—C12—C13	−0.5 (3)
C12—C3—C4—O3	−178.8 (2)	C10—C11—C12—C13	179.0 (2)
C2—C3—C4—C5	−177.7 (2)	C3—C12—C13—O1	179.4 (2)
C12—C3—C4—C5	1.8 (3)	C11—C12—C13—O1	−0.9 (3)
O3—C4—C5—C6	−2.7 (3)	C3—C12—C13—C14	−0.3 (3)
C3—C4—C5—C6	176.8 (2)	C11—C12—C13—C14	179.5 (2)
O3—C4—C5—C10	177.7 (2)	O4—C1—C14—C13	178.4 (2)
C3—C4—C5—C10	−2.9 (3)	C2—C1—C14—C13	−1.2 (3)
C10—C5—C6—C7	0.3 (3)	O4—C1—C14—C15	−2.6 (4)
C4—C5—C6—C7	−179.4 (2)	C2—C1—C14—C15	177.8 (2)
C5—C6—C7—C8	−1.1 (4)	O1—C13—C14—C1	−178.5 (2)
C6—C7—C8—C9	0.8 (4)	C12—C13—C14—C1	1.1 (3)
C7—C8—C9—C10	0.3 (4)	O1—C13—C14—C15	2.4 (3)
C8—C9—C10—C5	−1.1 (4)	C12—C13—C14—C15	−178.0 (2)
C8—C9—C10—C11	177.4 (2)	C16—O5—C15—O5'	−77.9 (18)
C6—C5—C10—C9	0.8 (3)	C16—O5—C15—C14	−168.5 (6)
C4—C5—C10—C9	−179.5 (2)	C16—O5'—C15—O5	77.2 (17)
C6—C5—C10—C11	−177.7 (2)	C16—O5'—C15—C14	172.5 (6)
C4—C5—C10—C11	2.0 (3)	C1—C14—C15—O5	8.4 (6)
C9—C10—C11—O2	1.1 (3)	C13—C14—C15—O5	−172.6 (6)
C5—C10—C11—O2	179.6 (2)	C1—C14—C15—O5'	−8.8 (6)
C9—C10—C11—C12	−178.4 (2)	C13—C14—C15—O5'	170.2 (6)
C5—C10—C11—C12	0.1 (3)	C15—O5—C16—O5'	77.8 (18)
C2—C3—C12—C13	−0.5 (3)	C15—O5'—C16—O5	−77.3 (17)
C4—C3—C12—C13	179.98 (19)		

### Hydrogen-bond geometry (Å, °)

**Table:**

*D*—H···*A*	*D*—H	H···*A*	*D*···*A*	*D*—H···*A*
O1—H1o···O2	0.85 (1)	1.79 (2)	2.557 (2)	150 (3)
O4—H4o···O5	0.84 (1)	1.77 (2)	2.546 (7)	152 (4)
O4—H4o···O5'	0.84 (1)	1.77 (2)	2.539 (7)	152 (4)
